# Cardiogenic Induction of Pluripotent Stem Cells Streamlined Through a Conserved SDF-1/VEGF/BMP2 Integrated Network

**DOI:** 10.1371/journal.pone.0009943

**Published:** 2010-04-01

**Authors:** Anca Chiriac, Timothy J. Nelson, Randolph S. Faustino, Atta Behfar, Andre Terzic

**Affiliations:** Marriott Heart Disease Research Program, Division of Cardiovascular Diseases, Departments of Medicine, Molecular Pharmacology and Experimental Therapeutics, and Medical Genetics, Mayo Clinic, Rochester, Minnesota, United States of America; Harvard Medical School, United States of America

## Abstract

**Background:**

Pluripotent stem cells produce tissue-specific lineages through programmed acquisition of sequential gene expression patterns that function as a blueprint for organ formation. As embryonic stem cells respond concomitantly to diverse signaling pathways during differentiation, extraction of a pro-cardiogenic network would offer a roadmap to streamline cardiac progenitor output.

**Methods and Results:**

To resolve gene ontology priorities within precursor transcriptomes, cardiogenic subpopulations were here generated according to either growth factor guidance or stage-specific biomarker sorting. Innate expression profiles were independently delineated through unbiased systems biology mapping, and cross-referenced to filter transcriptional noise unmasking a conserved progenitor motif (55 up- and 233 down-regulated genes). The streamlined pool of 288 genes organized into a core biological network that prioritized the “Cardiovascular Development” function. Recursive *in silico* deconvolution of the cardiogenic neighborhood and associated canonical signaling pathways identified a combination of integrated axes, CXCR4/SDF-1, Flk-1/VEGF and BMP2r/BMP2, predicted to synchronize cardiac specification. *In vitro* targeting of the resolved triad in embryoid bodies accelerated expression of Nkx2.5, Mef2C and cardiac-MHC, enhanced beating activity, and augmented cardiogenic yield.

**Conclusions:**

Transcriptome-wide dissection of a conserved progenitor profile thus revealed functional highways that coordinate cardiogenic maturation from a pluripotent ground state. Validating the bioinformatics algorithm established a strategy to rationally modulate cell fate, and optimize stem cell-derived cardiogenesis.

## Introduction

Pluripotent stem cells provide regenerative medicine with the broadest repertoire of developmental programs [1]–[7]. Diversity of stem cell derived cytotypes, each with discrete conditions underlying lineage differentiation, necessitates targeted strategies to harness tissue-specific progeny [8]–[13]. During differentiation of pluripotent stem cells, cardiac progenitors are embedded within heterogeneous populations of non-cardiac phenotypes [14]–[21]. Unsynchronized maturation of progeny, diverse patterns of biomarker expression, and variable exposure to growth factors or cytokine gradients confound the identification of a core transcriptional motif that governs cardiogenesis [22]–[26].

Mapping the cardiogenic blueprint, innate to pre-cardiac mesoderm, would offer a strategy to optimize cardiac lineage procurement from embryonic stem cells (ESCs). As an initial step, exposure of pluripotent ESCs to cardiopoietic factors was found to enrich genomic programs towards cardiac lineage commitment [27]. Characterization of cardiogenic progeny, derived *via* guided differentiation of the mesoderm through endodermal cues, extracted an ontological infrastructure that encompassed sarcomerogenesis, excitation-contraction coupling, and calcium handling suggesting a dynamic transcriptome that secures cardiogenesis [16]. In fact, a separate high-throughput bioinformatics approach, based on a distinctive cell surface biomarker signature during early cardiac differentiation, sorted cardiogenic precursors out of a pool of unconstrained pluripotent stem cell progeny [28]. The cardiac predisposition originating from these independent progenitor subpopulations implies a conserved cardiogenic gene network disguised by stochastic transcriptional noise within the pleiotropic stem cell background [29], [30].

To distill essential signaling components and prioritize pathways of cardiac differentiation across distinct cytotype fates would require an integrated, systems-based approach [31], [32]. Unbiased functional resolution of genome-wide transcriptional profiles may, to this end, provide a rational basis for targeted optimization of phenotypic output [33]–[35]. Here, cross-referencing a comprehensive pool of genome-wide transcripts within distinct progenitor platforms systematically identified the conserved components that secured cardiogenic commitment. Comparison of growth factor-guided and biomarker-sorted cardiogenic subpopulations filtered non-cardiac transcriptional noise to unmask a pre-cardiac blueprint that contained a core cardiac network with complementary hubs predicted to drive cardiogenesis. Targeting the prioritized collective axis of SDF-1/CXCR4, VEGF/Flk-1 and BMP2/BMPr was sufficient to augment *in silico*-postulated cardiac differentiation, and accelerate *in vitro* cardiogenesis. This strategy establishes a novel paradigm in which comparative refinement prioritized conserved tissue-specific targets across progenitor profiles, streamlining production of stem cell-derived pedigrees.

## Results

### Heterogeneity of pre-cardiac transciptome masks early lineage-specific pathways

**Figure 1:**
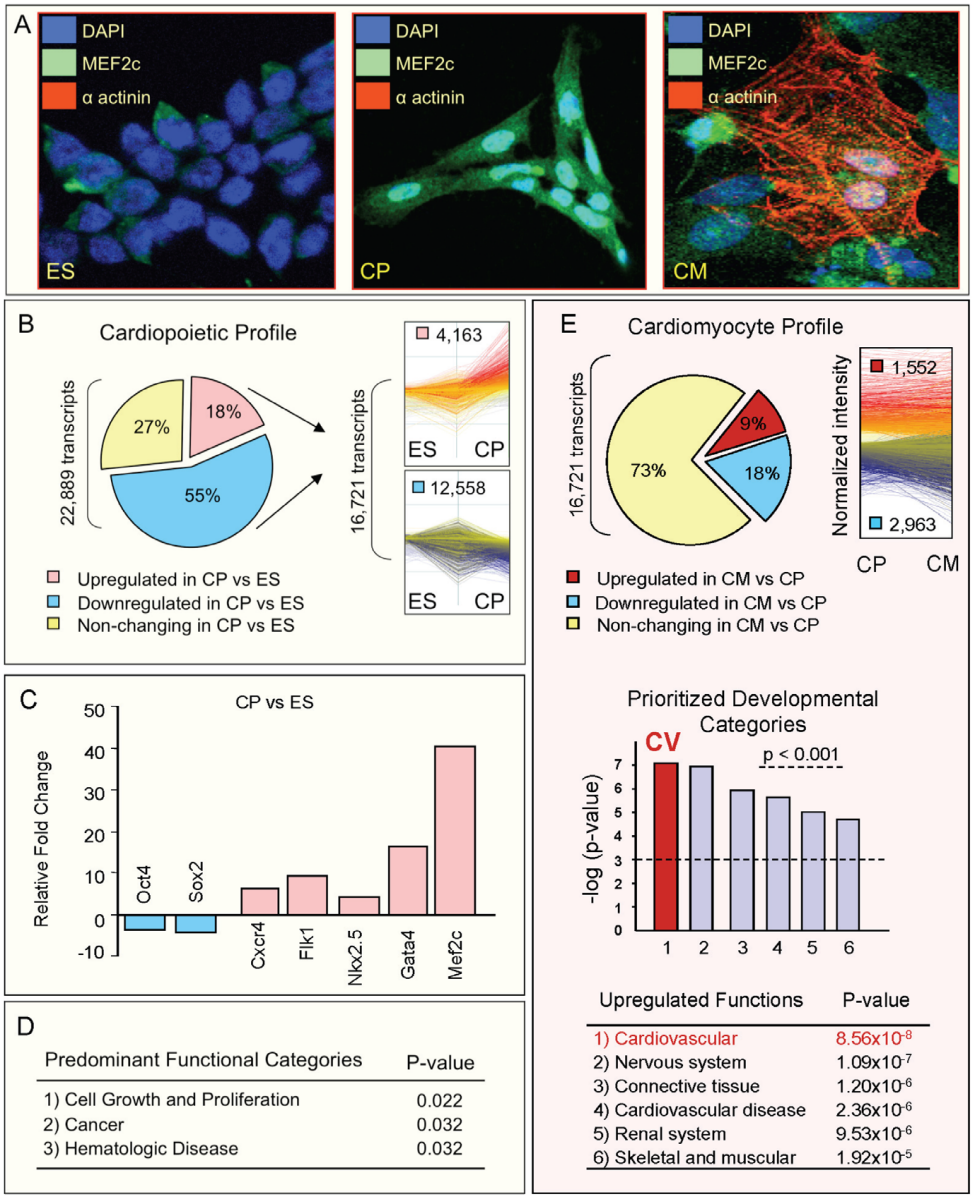
Heterogeneity of a primitive cardiac progenitor transcriptome precludes prioritization of cardiac pathways. (**A**) TNF-α guided differentiation of pluripotent embryonic stem cells (ES) facilitated generation of cardiopoietic (CP) progenitors, marked by nuclear internalization of Mef2C. Differentiated cardiomyocytes (CM) co-expressed nuclear Mef2C and sarcomeric α-actinin. (**B**) The cardiopoietic transcriptional profile encompassed 16,721 genes differentially expressed in CP versus ES. The signature CP transcriptome profile was defined here as a composite of significantly changed gene levels (≥20%, p<0.05) compared to the ES source. Blue, down-regulated genes; Red, up-regulated genes; Yellow, non-changing genes. (**C**) RT-PCR confirmed microarray predicted trends for selected transcripts. Blue, down-regulated genes; Red, up-regulated genes. (**D**) Ingenuity functional analysis prioritized generic developmental categories within the rich transcriptional profile of cardiopoietic (CP) cells. (**E**) Upper – Differentiated cardiomyocytes (CM) were distinguished from CP counterparts by 4,515 differentially expressed genes. Blue, down-regulated genes; Red, up-regulated genes; Yellow, non-changing genes. Lower – Within CM-specific transcripts, “Cardiovascular Development” (p = 8.56×10^−8^, highlighted in red in histogram) was prioritized among all other upregulated developmental programs.

Pluripotent stem cells within differentiating embryoid bodies, primed by the growth factor TNF-α for cardiogenic guidance [11], [27], produced enrichment in cardiac tissue (Figure 1). Within the heterogeneous cellular mixture (Figure 1A, left), guided pre-cardiac cells were characterized by an intermediate phenotype that included nuclear internalization of cardiac transcription factors (Mef2C, Nkx2.5) yet absence of cytoplasmic contractile proteins (Figure 1A, middle). Progenitor cells were enriched from day 7 beating embryoid bodies according to density gradient separation, and by day 12 evolved into differentiated cardiomyocytes that displayed co-staining of nuclear Mef2C with cytoplasmic α-actinin (Figure 1A, right). Genome-wide microarray analysis identified a cardiopoietic profile consisting of 16,721 differentially expressed transcripts (4,163 up- and 12,558 down-regulated) that distinguished the pro-cardiac cytotype from the pluripotent ground state (Figure 1B). Independently, quantitative RT-PCR of selected transcripts (*Oct4*, *Sox2*, *CXCR4*, *Flk-1*, *Gata4*, *Nkx2.5*, *Mef2C*) confirmed cardiogenic commitment within the progenitor subpopulation (Figure 1C). Ingenuity function analysis of the 4,163 progenitor-specific up-regulated transcripts, however, revealed broad ontologies, such as “Cellular Growth and Proliferation”, “Hematologic Disease” and “Oncogenicity”, without prioritization of lineage-specific pathways (Figure 1D). Transition to the more mature cardiomyocytes, which encompassed 4,515 differentially upregulated transcripts, was necessary to demonstrate cardiogenic pathways (Figure 1E). Thus, due to transcriptional noise of early stage progenitors, prioritization of functional signaling pathways was unable *a priori* to resolve the predicted cardiac phenotype.

### Biomarker selected progenitors segregate a lineage-specific transcriptome

**Figure 2:**
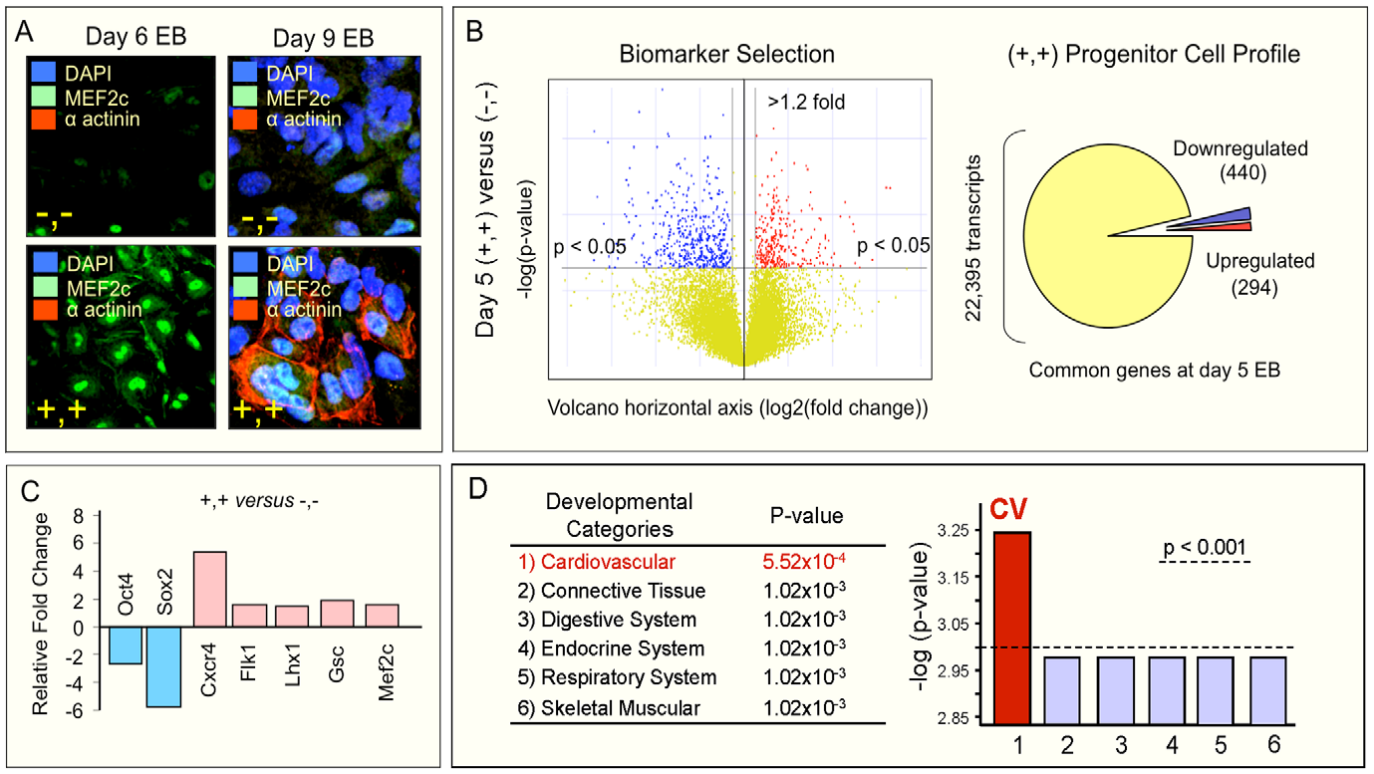
CXCR4/Flk-1 biomarker signature enriches for cardiac progenitors. (**A**) Sorted double positive (+,+) and double negative (−,−) cells were tracked for phenotypic differentiation in culture. Double positive cells displayed Mef2C nuclear localization at day 6 and expressed sarcomeric α-actinin at day 9 (lower panels). Double negative cells did not express markers of cardiac differentiation (upper panels). (**B**) Volcano plot analysis of gene expression data from double positive cardiac progenitors revealed significant differences compared to day 5 double negative cohorts. Red dots, differentially up-regulated transcripts (≥20% fold change, p<0.05); blue dots, down-regulated transcripts (≥20% fold change, p<0.05); yellow dots, shared transcriptome at day 5 embryoid bodies, independent of CXCR4/Flk-1 biomarker expression. (**C**) RT-PCR confirmed microarray predicted gene expression trends for selected transcripts. Blue, down-regulated, Red, up-regulated. (**D**) Ingenuity functional analysis of the double positive progenitor transcriptome revealed an ontologic prioritization (1) of “Cardiovascular Development” (p = 5.52×10^−4^), compared to other physiological systems (2–6).

A dual biomarker signature extracts a stage-specific cytotype according to cell surface expression of CXCR4/Flk-1 after 5 days of spontaneous differentiation from pluripotent stem cells [28]. Compared to CXCR4^−^/Flk-1^−^ counterparts lacking the ability to differentiate into cardiomyocytes, CXCR4^+^/Flk-1^+^ progenitors here expressed nuclear Mef2C at day 6 (Figure 2A, left) and co-expressed cardiomyocyte specific nuclear Mef2C and sarcomeric α-actinin at day 9 (Figure 2A, right). Genome-wide microarray analysis revealed a high degree of similarity between CXCR4^+^/Flk-1^+^ and CXCR4^−^/Flk-1^−^ subpopulations at day 5, yet the divergent gene expression profile represented more than 700 unique transcripts (Figure 2B). Validation of the microarray-predicted pro-cardiac predisposition in the CXCR4^+^/Flk-1^+^ subpopulation was obtained *via* a panel of pluripotency (*Oct4*, *Sox2*), endoderm (*Sox17*), mesoderm (*Goosecoid*, *Lhx1*), and cardiac-specific markers (*Gata4*, *Nkx2.5*, *Mef2C*, *Myocd*; Figure 2C). Ingenuity functional analysis of the 294 up-regulated and 440 down-regulated transcripts that distinguished CXCR4^+^/Flk-1^+^ from CXCR4^−^/Flk-1^−^ subpopulations identified an overt ontologic prioritization of “Cardiovascular Development” (p = 5.52×10^−4^; Figure 2D). Thus, a biomarker-selected subpopulation from spontaneously differentiated pluripotent stem cells identifies a pool of genes that non-stochastically integrate into a blueprint providing instructions for cardiac lineage-specification.

### Conserved gene expression profile extracts a cardiogenic network

**Figure 3:**
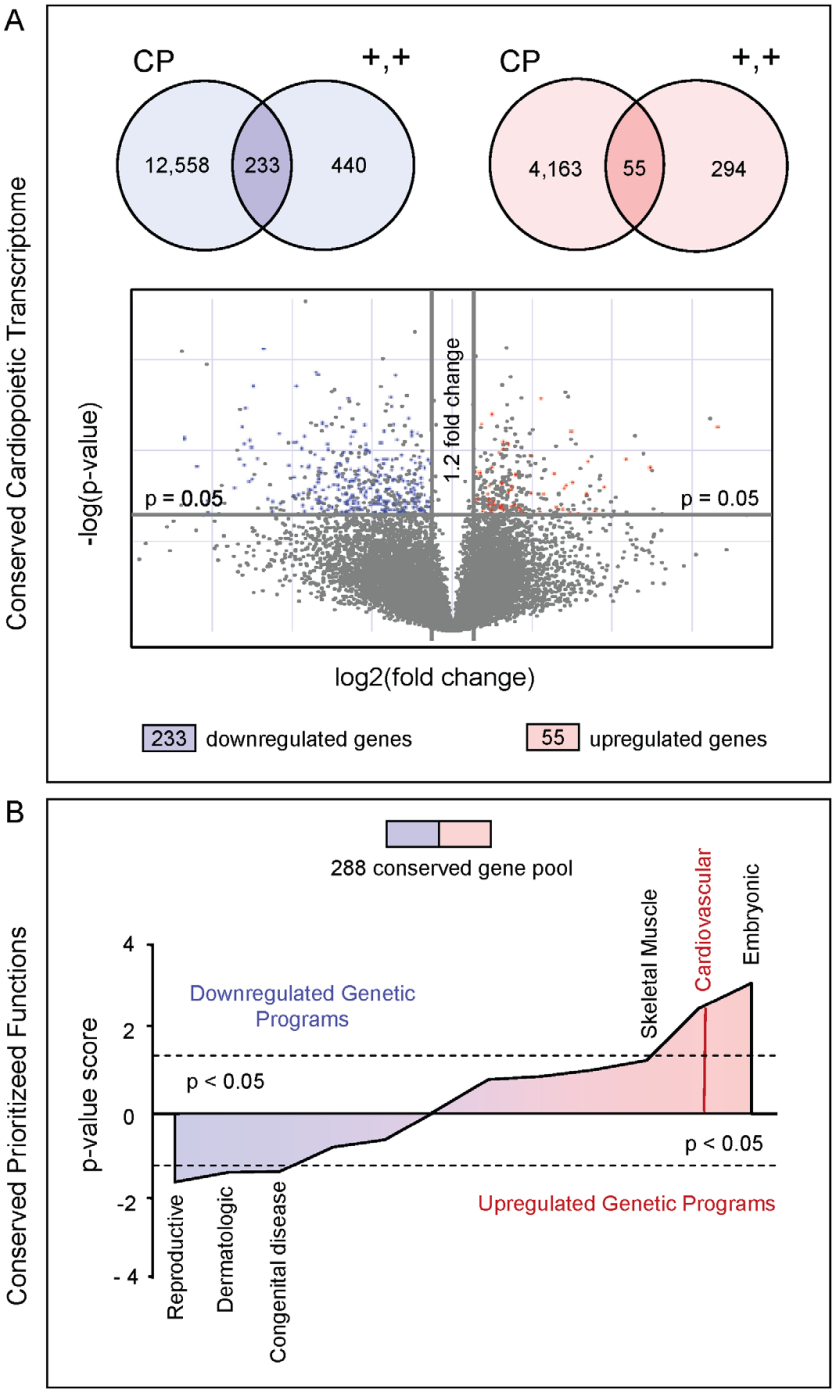
Transcriptome intersection of cardiopoietic and double positive progenitors uncovers a conserved cardiac signature. (**A**) Upper – Venn diagrams revealed a common transcriptome pool of 55 up-regulated and 233 down-regulated genes that represented similarly changing genes in cardiopoietic (CP) and double positive (+,+) cells. Lower – Volcano plot analysis of the double positive progenitor cell transcriptome (grey), overlaid with up-regulated (red dots) and down-regulated (blue dots) transcripts shared with CP. (**B**) *In silico* gene ontology analysis of the common progenitor transcriptome revealed persistent functional prioritization of “Cardiovascular Development”. Developmental categories are plotted according to a p-value score calculated to represent a weighted ontologic functional prioritization within the integrated 288 gene pool, i.e., p-value score  =  -log(p-value-Upregulated List) - (-log(p-value-Downregulated List). Red (positive y-axis) designates overall upregulated developmental programs within the integrated transcriptome, while blue (negative y-axis) designates overall downregulated developmental programs.

Based on two independent transcriptome profiles of growth factor guided (Figure 1) or stage-specific biomarker selected (Figure 2) progenitors, unbiased bioinformatic cross-referencing identified clusters of genes conserved during cardiogenic specification. Comparative analysis of progenitor profiles distilled a common roster of pro-cardiogenic genes using Venn diagrams (Figure 3A), with 55 up-regulated and 233 down-regulated transcripts relative to non-cardiogenic populations. Ingenuity function analysis further characterized the overrepresented developmental ontologies and signaling pathways encoded by this conserved gene expression profile, with an overall prioritization of “Cardiovascular Development” (Figure 3B). Restriction of progenitor gene expression profiles according to conserved identities streamlined the list of candidate genes by more than 70%, and strengthened the prioritization of cardiac developmental pathways.

### Prioritized hubs for cardiogenic induction

**Figure 4:**
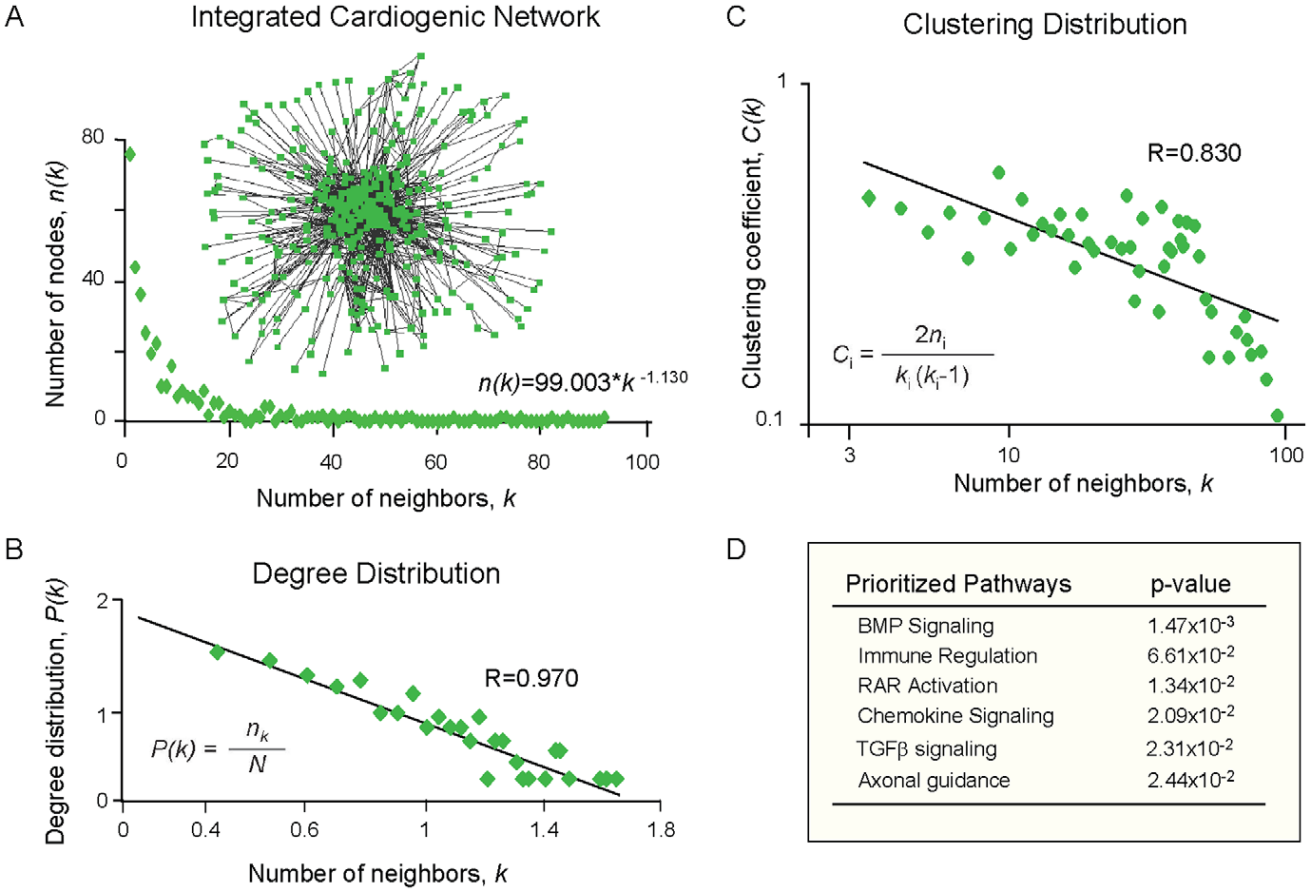
Conserved gene expression between progenitor transcriptomes extracts a network with quantifiable topology. (**A**) Ingenuity built an integrated cardiogenic scaffold of 363 molecules (i.e., network nodes) connecting the 288 progenitor shared genes and their direct, database predicted biological partners. Cytoscape interaction analysis decoded 1801 direct molecular relationships (i.e., network edges). X-axis represents the number of neighbors, *k*, for each network node. Y-axis, *n(k)*, represents the number of nodes with *k* neighbors and follows a power-law equation, with the majority of nodes having few connections and several highly connected nodes. The average calculated degree of the network, i.e., average number of neighbors for each gene (*k*) was 9.5. (**B**) The degree distribution, *P(k)*, established a linear correlation (R = 0.970) on the log-log scale with the number of neighbors, *k*, a feature characteristic of scale-free networks. (**C**) The clustering coefficient, *C(k)*, was calculated according to the inset formula, and correlated (R = 0.830) on a log-log scale with the number of neighbors, *k*. A linear log*C(k)* distribution denotes the hierarchical nature of the network. (**D**) The prioritized canonical pathways within the integrated network included BMP2, CXCR4/chemokine and TGFβ signaling.

**Figure 5:**
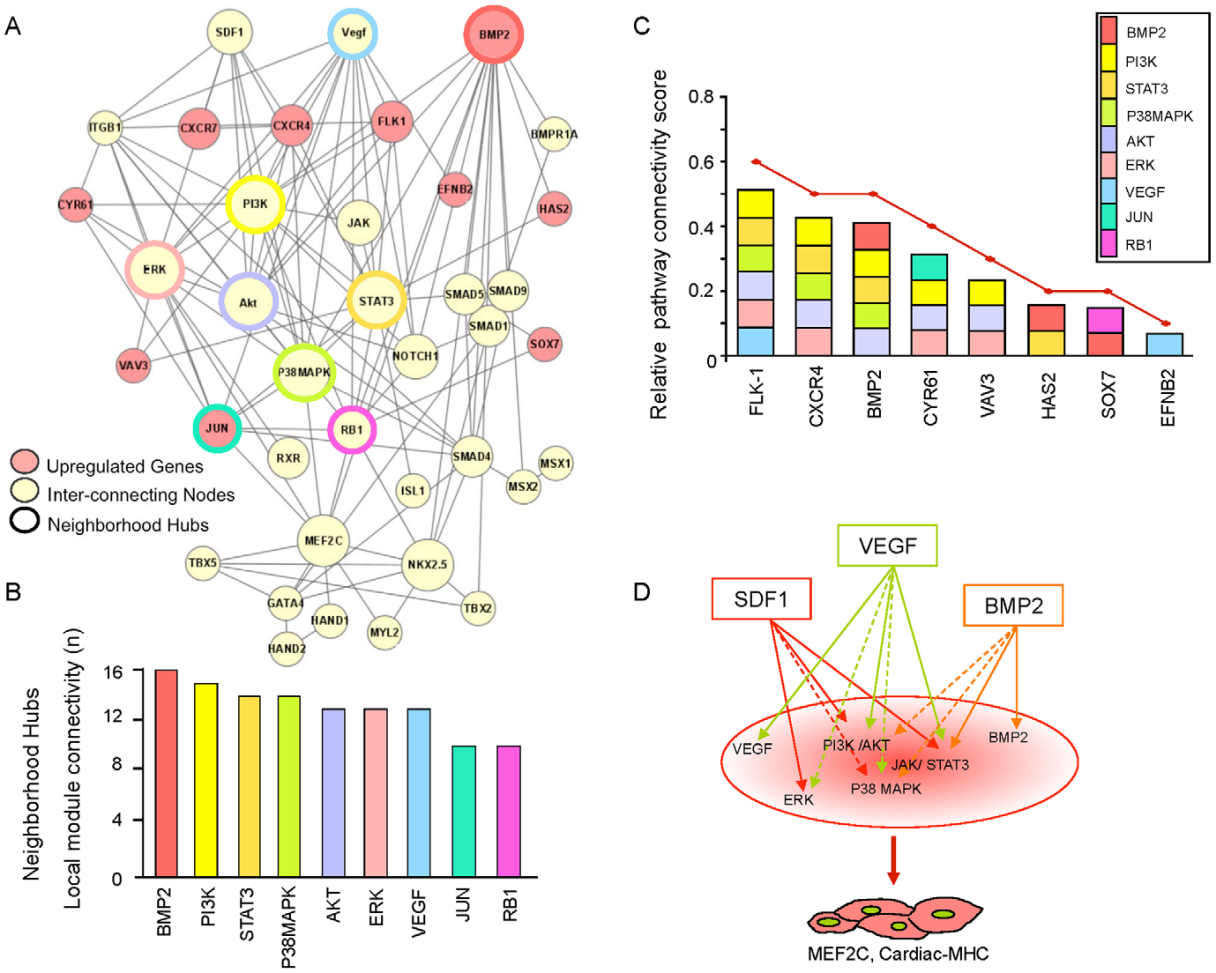
Prioritization of network hubs for cardiogenic induction. (**A**) A neighborhood of genes, cross-referenced with the Mouse Genome Informatics (MGI) knockout-based cardiac phenotype database, was independently established by Ingenuity as the most prioritized sub-network of the integrated cardiogenic framework. Nodes in the network are pink (upregulated pro-cardiac genes) or yellow (database-predicted direct interacting genes). Hubs are defined as highly connected nodes (genes) outlined in the network by colored circles. (**B**) Local module connectivity was defined for each of the network hubs as the total number of node connections they establish. (**C**) A relative pathway connectivity score (red, line graph) was calculated for the upregulated pro-cardiac genes, defined as the ratio of the number of hubs each cardiac gene is connected to over the total number of hubs available in the sub-network. (**D**) The integrated signaling model predicts the behavior of the network in the context of a triple treatment that would collectively target all integrating signaling highways. Co-stimulation of the SDF-1/CXCR4, VEGF/Flk-1 and BMP2/BMP2r axes is predicted to simultaneously activate all network hubs and empirical cardiac genes. Biological output will be quantified *via* nuclear transcription factors (i.e., Mef2C) and reporter assays (cardiac-MHC–Lac Z) as surrogates for *de facto* cardiogenic specification.

Biological networks exhibit quantifiable architectural characteristics [36]. Here, the conserved transcriptome formed an integrated cardiogenic network (Figure 4A) that exhibited mathematical property distributions attributed to scale-free (Figure 4B), and hierarchical (Figure 4C) network topologies, consistent with non-stochastic physiological systems. Ingenuity Pathway Analysis predicted and quantified preferential signaling pathways within this progenitor network. BMP2 signaling was overall prioritized (p = 1.47×10^−3^), followed by regulation of innate immunity (p = 6.61×10^−2^), retinoic acid receptor activation (p = 1.34×10^−2^), CXCR4-dependent chemokine signaling (p = 2.09×10^−2^), and TGFβ1 signaling (p = 2.31×10^−2^; Figure 4D). Based on this conserved cardiogenic network, an unbiased recursive bioinformatics algorithm prioritized a neighborhood of genes independently associated with cardiovascular developmental ontologies and centered on highly connected hubs, including BMP2, PI3K, STAT3, P38MAPK, Akt, ERK, VEGF, Jun and Rb1 (Figure 5A). Transcripts upregulated and related to the hubs, namely BMP2, CXCR4, CXCR7, EFNB2, Flk-1, HAS2, Sox7, CYR61, VAV3 and Jun, were validated in each progenitor population, and independently linked to cardiovascular specification or cardiac pathophenotypes based on the Mouse Genome Informatics Database. Furthermore, this empirical gene cluster was separately identified by Ingenuity as the predominant sub-network of the integrated transcriptome, with an overall signaling prioritization related to “Cardiovascular Development” (Figure 5A). Hub inter-relationships were quantified based on local module connectivity, and calculated as the total number of direct gene connections representing a weighted importance of each signaling relay in cardiac differentiation pathways (see Materials and Methods). In this way, an order of hub connectivity was extracted with nine hubs providing the integrated scaffold of the cardiac neighborhood (Figure 5B). Hub interactions were mapped for each of the upregulated transcripts, and revealed that CXCR4/SDF-1, Flk-1/VEGF and BMP2/BMPr were sufficient to collectively hit all nine hubs of the network (Figure 5C). Thus, an integrated network, conserved across two cardiogenic cytotypes, and an unbiased systems biology approach streamlined the minimal triad of ligand/receptor pairs hypothesized *in silico* to simultaneously activate pro-cardiac pathways (Figure 5D).

### Targeted enhancement with SDF-1/VEGF/BMP2 promotes cardiogenic output from pluripotent stem cells

**Figure 6:**
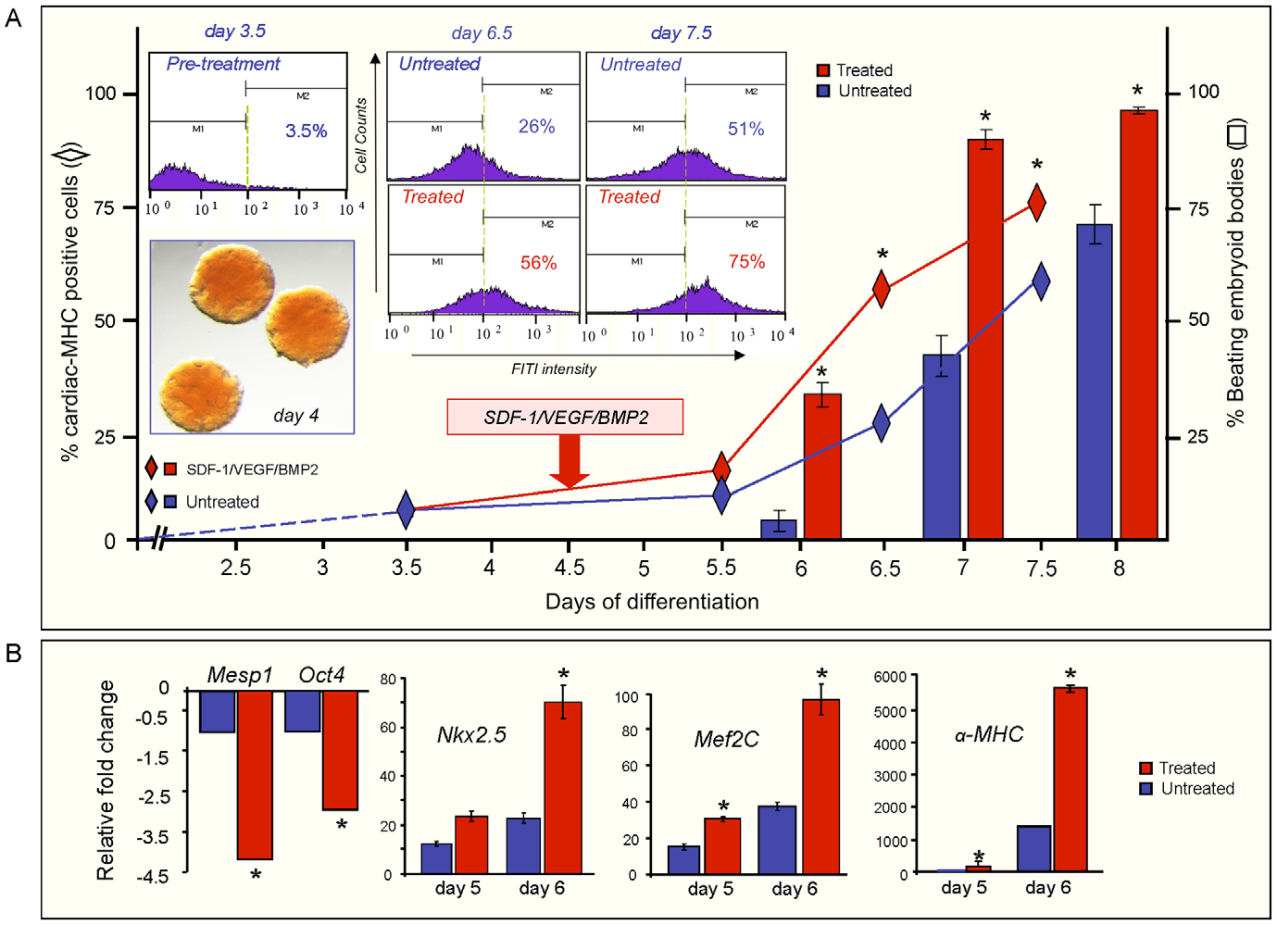
Targeted treatment with SDF-1/VEGF/BMP2 increases cardiogenic yield. (**A**) α-MHC R1 embryonic stem cells were differentiated by the hanging drop method. Baseline embryoid body (EB) beating activity (columns) and FACS-gal α-MHC positive cell embryoid body composition (diamonds) were quantified (blue). Floating EBs (inset) transferred to gelatinized 6 well plates in the evening of day 4 were treated with SDF-1 (100 ng/mL), VEGF (10 ng/mL), and BMP2 (10 ng/mL) or differentiation media alone at Day 4.5. EBs were collected each day for FACS-gal analysis, RT-PCR and X-gal staining. Beating activity was recorded daily for each treatment condition for ∼300 EBs. Beating activity is represented by columns (blue for baseline, red for treatment), % cardiac-MHC-Lac Z positive cells are represented by diamonds (blue, red respectively). (**B**) RT-PCR analysis for selected pluripotency (*Oct4*), pre-cardiac mesoderm (*Mesp1*) and canonical cardiac (*Nkx2.5*, *MEF2C*, cardiac-MHC) transcripts. Red bars represent treatment with SDF-1, VEGF and BMP2; blue bars represent the baseline, untreated condition.

Derived from embryoid bodies, fluorescence activated flow-sorting analysis of individual cells revealed a baseline profile of persistently low levels of cardiac-myosin heavy chain (MHC) expression during the first 5 days of progenitor differentiation (3.5% positive cells at day 3.5), followed by an induction at day 7 (∼26% positive cells) that reached a plateau (∼50% positive cells) at day 8 (Figure 6A). Treatment with SDF-1, VEGF and BMP2 induced significant enrichment (50% increase over baseline at days 6.5 and 7.5) in cardiac-MHC expressing cells in treated *versus* untreated embryoid bodies (Figure 6A). Moreover, targeted treatment with SDF-1, VEGF and BMP2 increased embryoid bodies beating activity as early as day 6 from 5% to 30%. The cardiogenic benefit was sustained throughout differentiation, with targeted treatment maximizing beating activity by day 8. Furthermore, treatment of day 5 embryoid bodies led to significant down-regulation of pluripotent (*Oct4*) and early pre-cardiac mesoderm (*Mesp1*) markers within 12 h of treatment, compared with untreated controls (Figure 6B, left). The treatment effect was followed by a significant induction in gene expression of cardiac transcription factors (*Nkx2.5*, *Mef2C*) and contractile proteins (cardiac-MHC), not observed in the untreated counterparts (Figure 6B, right). In treated progeny, RT-PCR demonstrated increased cardiac markers, such that expression kinetics coincided with beating activity. Therefore, functional activity, native gene expression, and induction of a cardiac reporter system corroborated the cardiogenic influence of applied treatment as predicted *in silico*.

**Figure 7:**
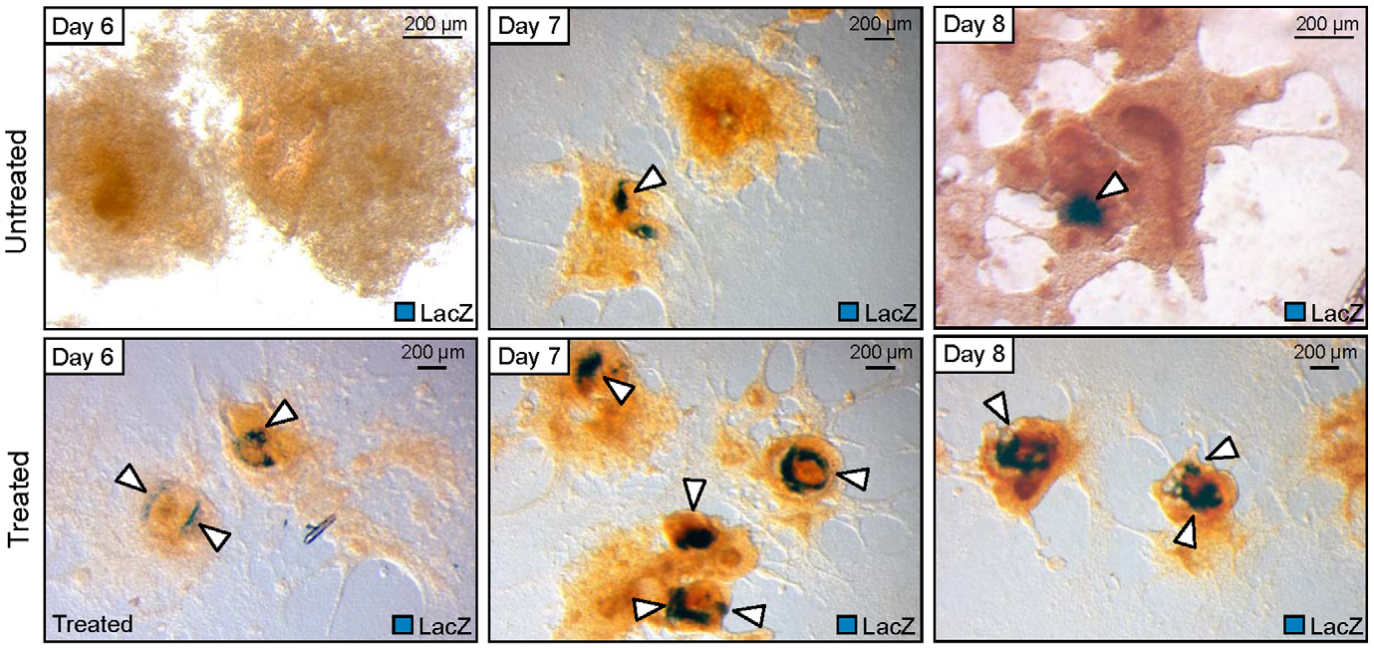
Augmentation of cardiac phenotype from pluripotent stem cells. X-gal stained embryoid bodies show early enrichment in cardiac-MHC-LacZ expressing areas (blue staining) after 2 days of treatment (Day 6). On subsequent days, treated EBs consistently showed upregulation in cardiac-MHC-LacZ expressing areas compared to untreated controls. Arrows indicated cardiac-MHC-LacZ expressing areas.

Individual embryoid bodies treated with SDF-1, VEGF and BMP2 consistently demonstrated no observable difference compared to untreated counterparts during early stages of differentiation, yet as early as day 6 exhibited an increase in cardiac-MHC promoter expression, with no corresponding LacZ reporter staining observed in the untreated control populations (Figure 7). Areas positive for cardiac-MHC/LacZ expanded on day 7, and embryoid bodies demonstrated >50% of cell mass expressing cardiac-MHC by day 8. Interestingly, embryoid bodies treated with the SDF-1, VEGF and BMP2 cocktail reliably produced a distinctive LacZ expression pattern with a ring-like shape or a linear alignment of α-MHC positive cytotypes in contrast to the random distribution of cardiogenic progeny in untreated controls. Furthermore, quantified increases in contractile foci were observed in SDF-1/VEGF/BMP2-treated embryoid bodies compared with untreated controls. Thus, targeted modulation of prioritized cardiogenic hubs exerts a quantifiable biological output in differentiating stem cells validating a rational approach to optimize cardiogenic induction from a pluripotent source and suggesting a strategy to position cardiac progenitors within tissue constructs according to chemokine gradients.

## Discussion

Heterogeneity of gene expression in differentiating pluripotent stem cells obscures the resolution of fate-specific developmental networks that initiate cardiac specification during embryonic organogenesis [37]–[39]. Here, bioinformatic refinement of a conserved transcriptome, cross-referencing two populations of stem cell-derived cardiac progenitors, sufficiently reduced platform-specific background noise to extract a robust pro-cardiogenic network from precursors captured *en route* to cardiomyocyte differentiation. Restricting analysis to the common pool of progenitor genes synchronized “Cardiovascular Development” within a streamlined ontological infrastructure, and revealed identities of prioritized network hubs that promoted pluripotent stem cell-derived cardiogenesis. Accordingly, targeted treatment with SDF-1, VEGF, and BMP2 was designed to activate predicted network hubs, a strategy that effectively accelerated cardiac determination. In fact, this rationally designed therapy, uncovered by genome-wide screening, demonstrated significant induction of *Nkx2.5*, *Mef2C*, cardiac-*MHC*, and enhanced beating activity from pluripotent progenitors. Thus, recursive bioinformatics analysis of discrete stem cell-derived progeny fostered deconvolution of a signature sub-network that contained ontologically aligned hubs responsive to pro-cardiac modulation, establishing a tailored approach to optimize cardiogenic yield from a pluripotent source.

Simultaneously active gene networks in differentiating stem cells create dynamic heterogeneities that lead to a spectrum of cellular phenotypes, a basis for transcriptional noise [29]. Heterogeneity reflects metastable states of a slowly fluctuating transcriptome that encodes distinct and reversible cytotypes which prime a pluripotent background for single lineage commitment and govern the stochastic process of cell fate decision [30]. In principle, signaling stimuli applied to a dynamic transcriptome could shift developmental equilibria towards progenitor states thereby increasing the probability of cardiogenic specification *versus* alternative developmental programs [30], [40]. Partial modulation of necessary signaling pathways *in vitro* may lead to inconsistent outcomes that plaque differentiation protocols based on random inclusion criteria. An unbiased approach to reveal a comprehensive and integrated strategy may thus offer a higher degree of reproducibility. Here, a cardiac specific gene regulatory network provided a molecular fingerprint of mitotically active, yet lineage committed cardiac progenitors, that facilitated selective targeting and clonal expansion of a therapeutically relevant pro-cardiac population.

As developmental programs for multiple lineages are simultaneously activated in the differentiating embryoid body, a heterogeneous mixture of cytotypes emerges that confound cardiac lineage-specific contributions [41]–[44]. Strategies to enrich cardiac progenitors are thus required to harness the regenerative potential of pro-cardiac progeny while eliminating the risks of uncontrolled teratogenic expansion [8], [13], [24], [41]. Our initial strategy of endoderm-based recombinant growth factor-guided stimulation of embryonic stem cells extracted an intermediate population of cardiopoietic progenitors that were enriched according to the limitation of density gradients [27]. Genome-wide expression analysis solved this pro-cardiac phenotype in relationship with *bona fide* cardiomyocytes, and evidenced the underlying activated pathways of pro-cardiac to cardiac determination [16]. Here, a cardiopoietic transcriptional profile of 16,721 genes uniquely identified the intermediate cardiac phenotype and offered the initial blueprint for candidate gene discovery of progenitor specific biomarkers. The inherent stochasticity associated with large pools of genes was found to increase the transcriptional noise in cardiopoietic progenitors, and preclude prioritization of signaling axes for regulation of cardiogenic output. Only general developmental categories, such as “Cell Growth and Proliferation”, were associated with this parental, density-enriched cardiopoietic population that inevitably contained confounding cytotypes. A subsequent strategy capitalized on the uncovered cardiopoietic-specific cell surface biomarkers and flow sorting strategies to enrich a homogeneous population of CXCR4^+^/Flk-1^+^ double positive cardiac progenitors [28]. Indeed, genome-wide analysis of this synchronized clonal population revealed less variability than cardiopoietic progenitors and a compact transcriptome of 734 genes that collectively encoded the required pathways for cardiogenic specification. Cross-referencing gene expression profiles of double positive progenitors with the parental intermediate phenotype further reduced transcriptional noise, and streamlined 288 conserved cardiogenic transcripts. Therefore, unbiased recursive refinement of pro-cardiac transcriptional profiles restricts genomic complexity from over 16,000 genes in cardiopoietic cells to 734 genes in double positive progenitors, and ultimately 288 genes in the conserved transcriptome, while progressively increasing pro-cardiovascular ontologic prioritization and unmasking potentially necessary and sufficient hubs for cardiogenic activation.

The ability of two unique cellular subpopulations to acquire a common cardiac endpoint implies requisition of core gene networks that collectively guide a heterogeneous transcriptome to engage fate-specific programs. Intersection of discrete transcriptomes, from progenitors with a common fate, thus filtered transcriptional noise and facilitated elucidation of persistent molecular elements that comprise a stabilized transcriptome essential for ultimate cardiogenic determination. Furthermore, the conserved cardiac pedigree integrated and organized as a network with quantifiable, biologically relevant properties [36]. This ontologic infrastructure delineated a cardiac neighborhood encompassing up-regulated progenitor-specific genes with a pro-cardiac phenotype, cardiovascular transcription factors, and interconnecting hubs (SDF-1/CXCR4, VEGF/Flk-1, BMP2/BMP2r) that are relays to major canonical signaling highways. Thus, the distilled cardiac and vascular sub-network rationally targeted pathways for enhancement of cardiovasculogenesis.

Network architecture deconvolution predicted the necessary combination of signaling axes to stimulate prioritized hubs, and thus activate in a targeted manner the preferential signaling highways for cardiogenic specification. Bioinformatic network predictions were validated when SDF-1/VEGF/BMP2-treated embryoid bodies displayed precocious expression and sustained up-regulation of cardiac transcription factors, beating activity and cardiac cell yield, compared to untreated controls. Although stem cell cardiogenesis has previously been enhanced with BMP2 [45], [46] and VEGF [47], [48], stromal derived stem cell factor (SDF-1) has yet to be demonstrated as a pro-cardiogenic cytokine on pluripotent stem cells. In line with the present study, initial evidence for a role of SDF-1 in cardiogenesis was postulated by the observed cardiac phenotype in knockout transgenic strains [49], and by the established role of the CXCR4/SDF-1 signaling axis in cardiac regeneration after myocardial infarction [50]. Furthermore, from pluripotent stem cells, pre-cardiac expression of the SDF-1 receptor CXCR4, in combination with Flk-1, has demonstrated the ability to isolate cardiac precursors [28]. Although not the focus of this *in vitro* analysis, the biological and possible clinical significance of SDF-1/CXCR4 axis alone or combination with other soluble factors may provide a relevant target to not only promote progenitor cell localization to the most appropriate niche environment but also significantly contribute to cardiac-specific differentiation within the injured heart. Therefore, the bioinformatics prediction of the unique integration of hubs encompassing SDF1/CXCR4, VEGF/FLK-1, and BMP2/BMPr was grounded by independent evidence, and was here demonstrated for the first time as a potent signaling triad for enhanced cardiogenesis with potential for translation into biomedical applications.

Embryoid body differentiation is a versatile model system to dissect molecular mechanisms underlying cardiac fate decision according to genomic, proteomic and/or metabolomic profiling [16], [51]–[53]. Successful translation of pluripotent stem cell technology is contingent upon maximizing efficacy of lineage-specification and minimizing risk of dysregulated oncogenic growth. The therapeutic applicability of stem cell cardiogenic platforms is critically dependent upon isolation of homogeneous populations of cardiac progenitors to optimize myocardial repair and minimize risks of *in situ* teratogenic transformation [15], [27], [54], [55]. High volume generation of stem cell-derived cardiomyocytes from *in vitro* procurement has proven technically challenging, and subject to random selection or inter-platform variability [4], [8], [12], [41]. Deconvoluting transcriptional hierarchy pertaining to cardiovascular commitment, regardless of procurement strategy, is a feasible approach as demonstrated herein to extract essential factors required for cardiogenic differentiation from various stem cell backgrounds. Furthermore, *in silico* streamlining allowed ontological alignment and prioritization of pro-cardiac signaling axes, otherwise masked by platform heterogeneity, and enabled targeted enhancement. Overall, the present data demonstrate the biological relevance of a conserved progenitor transcriptome as a dynamic tool to prioritize essential developmental highways, and predict molecular targets for rational control of organogenesis. Direct targeting of hubs supporting cardiogenic specification, activates preferential pathways and elicits a quantifiable biologic output in embryoid bodies as evidenced by pro-cardiogenic synchronization of progeny derived from pluripotent stem cells, advancing traditional probabilistic methods into a novel deterministic strategy for lineage induction.

## Materials and Methods

### Embryonic Stem Cell Cardiopoiesis

Murine embryonic stem cells, CGR8 and R1 [27], [28], were maintained in Glasgow's Minimum Essential Medium (BioWhittaker-Cambrex) supplemented with pyruvate and L-glutamine (Cellgro), non-essential amino acids (Mediatech), β-mercaptoethanol (Sigma-Aldrich), 7.5% fetal calf serum (FCS) (Invitrogen) and leukemia inhibitory factor (LIF) (ESGRO, Chemicon). Stem cells were differentiated into embryoid bodies using the hanging-drop method [56], [57] in differentiation media supplemented with 20% FCS and TNF-α (Invitrogen). Dual interface Percoll gradient (Invitrogen) was used to separate sarcomere-rich, high density cardiomyocytes from lower density, sarcomere-poor cardiopoietic precursors from day 7 embryoid bodies that were guided towards cardiogenic pathways according to TNF-α treatment [27]. Embryonic stem cell-derived progeny were fixed in 3% paraformaldehyde, permeabilized with 1% Triton X-100, and immunostained with antibodies specific for Mef2C (1∶400, Cell Signaling) Nkx2.5 (1∶150, Santa Cruz) and sarcomeric protein α-actinin (1∶1,000, Sigma-Aldrich) along with DAPI staining to visualize nuclei [27]. Microscopy was performed using an LSM 510 laser scanning confocal microscope (Carl Zeiss).

### Flow Sorting for Biomarker Selected Cardiac Progenitors

Embryoid bodies after 5 days of differentiation were washed in phosphate buffered saline (PBS) and dissociated using non-enzymatic dissociation buffer (Invitrogen) for 10 min at 37°C. Aggregates were triturated to obtain single cell suspensions. Derived cells were spun down at 1,000 *g* for 5 min and resuspended in propagation media (7.5% FCS) for 10 min to allow cells to recover. Cells (2×10^7^) obtained from initial aggregates were collected and immuno-stained for Flk-1 and CXCR4 biomarker expression [19], [28]. Cells were washed with PBS and resuspended in 1 ml PBS which contained goat-CXCR4 antibody (1∶150, Abcam), placed on ice for 30 min incubation, followed by single wash with 10 ml PBS. Secondary anti-goat Alexa 488 (1∶500, Molecular Probes, Invitrogen) and phycoerythrin (PE)-conjugated primary antibody for Flk-1 (1∶200, BD Biosciences) were incubated on ice for 30 min followed by single 10 ml PBS wash. Cells were isolated using a FACS Aria SE flow cytometer (BD Biosciences). Alexa-488 was excited with a 488 nm argon laser and detected through a 530/30 nm bandpass filter. PE was excited with the 488 nm laser line and detected through a 575/26 nm bandpass filter. Forward and side scatter parameters were used to gate viable cell population sorted into subpopulations.

### RNA Isolation and Microarrays

Total RNA was isolated using the Micro-to-Midi Total RNA Purification System (Invitrogen) or a Qiagen protocol using a combination of gDNA Eliminator and RNeasy columns. Three independent biological replicates were obtained for each condition, with a total of twelve biological samples for guided cardiogenesis and six biological samples for biomarker selection. Double stranded cDNA and labeled complementary cRNA were obtained from isolated RNA with the latter hybridized to the Mouse 430 2.0 GeneChip (Affymetrix). Gene Chips were scanned with an argon-ion laser and data visualized using the Affymetrix Microarray Suite 5.0 software.

### Gene Expression Analysis

Microarray gene expression raw data were MIAME compliant, and were deposited with the Gene Expression Omnibus database (Guided cardiogenesis, Accession number: GSE6689; Biomarker selection, Accession number: GSE20841). Analysis was performed using the GeneSpring GX 7.3.1 software (Agilent Technologies). All probe sets were filtered according to chip-specific background noise, and genes expressing signals below threshold were removed [34]. Quality filtering was performed according to an established flag value, with values that are Present (P), Marginal (M) or Absent (A) assigned to the marker. For guided cardiopoiesis, probe sets from all conditions were normalized to gene expression levels at pluripotent embryonic stem cell stage. For biomarker selection, probe sets from CXCR4^+^/Flk-1^+^ and CXCR4^−^/Flk-1^−^ cells were normalized to gene expression levels from CXCR4^−^/Flk-1^−^ hybridized arrays. To ensure that only genes with significant transcriptional changes during cardiogenesis are selected, all probe sets were filtered according to a flag value of Present or Marginal in at least two out of three replicates for one experimental condition. A filter on volcano plot was applied to identify significant changes in gene expression (>1.2 fold, p<0.05) in cardiopoietic progenitors compared to pluripotent embryonic stem cells, and in CXCR4^+^/Flk-1^+^ cells compared to their double negative counterparts.

### Quantitative Gene Expression

cDNA was synthesized from total RNA samples using SuperScript III First-Strand Synthesis System (Invitrogen). Real time PCR was performed using a standard TaqMan PCR kit protocol on an Applied Biosystems 7900HT Sequence Detection System (Applied Biosystems) as described [16], [19], [28]. The 50 µl PCR reaction mixture included 3 µl RT product, 25 µl TaqMAN Universal Master Mix (Applied Biosystems), 19.5 µl RNase-free water and 2.5 µl TaqMan Gene Expression Assays (pre-designed, pre-optimized probe and primer sets for each gene of interest). TaqMan Gene Expression assays contain 2 unlabeled PCR primers (900 nM each final concentration) and 1 FAM dye-labeled MGB probe (250 nM final concentration). Reactions were incubated in 96-well plates at 95°C for 10 min, followed by 40 cycles of 95°C for 15 s and 60°C for 1 min. The threshold cycle (Ct) was defined as the fractional cycle number at which fluorescence passes detection threshold. Ct values were subsequently converted into relative fold changes determined using the 2^−ΔΔCT^ method, normalized to *Gapdh* (P/N 435,2662-0506003). Genes representative for pluripotency, gastrulation and cardiogenesis were included in the analysis, such as *Oct4* (Mm00658129_gH), *Sox2* (Mm00488369_s1), *Cxcr4* (Mm01292123_m1), *Flk1* (Mm00440099_m1), *Nkx2.5* (Mm00657783_m1), *Gata4* (Mm00484689_m1), *Lhx1* (Mm00521776_m1), *Gsc* (Mm00650681_g1), *Mef2c* (Mm01340839_m1), *Mesp1* (Mm00801883-g1), and *α-MHC* (Mm00440347-gH).

### Bioinformatic Function Analysis

Venn diagrams within GeneSpring were used to identify overlapping transcripts with similar expression trends in both guided cardiopoietic and biomarker selected progenitors, and two lists were generated - commonly upregulated and commonly downregulated genes. Transcriptional profiles were functionally analyzed using the Ingenuity Pathway Analysis Software IPA 7.0 (www.ingenuity.com). Intersecting gene lists consisting of concordantly up- or down-regulated transcripts in progenitor populations were subsequently analyzed. Based on the curated Ingenuity Knowledge Database and using a right-tailed Fisher's Exact Test, overrepresented functions and pathways associated with intersecting gene lists were identified and the significance of association (p-value) was calculated based on the probability of pathway assembly from a random set of genes of the same size as the input list.

### Network Modeling and Topology Analysis

Network building tools available within Ingenuity were employed for bioinformatic modeling of networks [58], [59]. The Institute for Systems Biology Cytoscape 2.6.0 software (http://www.cytoscape.org) was applied to Ingenuity-prioritized networks to provide data regarding network topology in addition to visualizing relationships [11]. Direct molecular interactions were decoded using the Cytoscape visualization algorithm and basic mathematical properties, such as degree distribution (Equation 1) and clustering coefficient (Equation 2) were computed via Network Analyzer 2.6.1 (Max Planck Institute, Computational Biology and Applied Algorithmics, Saarbrücken, Germany, http://med.bioinf.mpi-inf.mpg.de/netanalyzer), as described [11], [60].
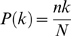



Equation 1. Degree distribution *P(k)*, where *n_k_* indicates number of nodes with *k* number of connections, over the total number of nodes, *N*.
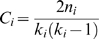



Equation 2. Clustering coefficient *C*_i_, where *n*_i_ represents the number of edges, or connections, adjacent to node (i), and *k*_i_ the number of nodes connected to node (i).

### Hub Prioritization

Knockout phenotypes associated with conserved gene pools were further investigated through bioinformatic mining of the Ingenuity and Mouse Genome Informatics Databases (www.informatics.jax.org). Within network clusters, inter-molecular relationships were deconstructed and quantified based on local module connectivity (n, n = number of direct gene connections) to weigh hubs according to neighborhood prioritization. A relative pathway connectivity score was used to rank upregulated pro-cardiac transcripts according to the degree of connectivity *via* hubs to highways of cardiogenic specification (Equation 3). 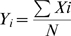



Equation 3. Relative pathway connectivity. A connectivity score, *Y_i_*, was calculated for upregulated pro-cardiac transcripts, defined as the ratio of total number of hubs connected to node *i*, *x_i_*, divided by the total number of hubs available in the sub-network, *N*.

### Cardiogenic Induction from Pluripotent Stem Cells

αMHC-lacZ R1 embryonic stem cells that express a cassette containing the αMHC promoter upstream of β-galactosidase cDNA were used to monitor acquired cardiogenesis, as previously described [61], [62]. Stem cells were maintained in Glasgow's Minimum Essential Medium (BioWhittaker-Cambrex) supplemented with pyruvate and L-glutamine (Cellgro), non-essential amino acids (Mediatech), β-mercaptoethanol (Sigma-Aldrich), 15% fetal calf serum (FCS) (Invitrogen) and leukemia inhibitory factor (LIF) (ESGRO, Chemicon). Embryoid bodies were generated according to the hanging drop method with or without treatment on day 4.5 with SDF-1 (100 ng/mL, provided by Dr. Brian Volkman, Medical College of Wisconsin, Milwaukee, Wisconsin [63], VEGF (10 ng/mL, R&D Systems; [46], [64]) and BMP2 (10 ng/mL, R&D Systems; [45], [46]).

### FACS Quantification of Cardiac-MHC Expressing Cells

FACS-Gal analysis was performed by FDG (fluorescein di-β-d-galactopyranoside) loading and FACS analysis [65]. Each day, embryoid bodies were collected, triturated with enzyme-free cell dissociation buffer (Invitrogen), and resuspended in ice cold HBSS+ buffer (1x HBSS, 2% Fetal calf serum, 10 mM Hepes buffer (pH 7.2), 1% Penicillin/Streptomycin) at 1×10^6^ cells/mL. Cellular samples (100 µL) and equal volumes of FDG staining aliquots (1∶10 dilution of the 20 mM FDG stock solution) were preheated at 37°C for 10 min. FDG was loaded onto cells and the solution was incubated at 37°C for 1 min before transfer into 15 mL Falcon tubes containing 2 mL ice cold HBSS+ buffer. The incubation continued on ice for 1.5 h allowing accumulation of the fluorescent product (FITC). Samples were analyzed fresh with a Becton Dickinson FACSscan using a 488 nm pre-excitation laser and a fixed FITC detection filter. Forward and side scatter parameters were used to gate the viable cell population. Unstained cells were used as a negative control for FITC gating, and the percentage of cardiac-MHC positive cells in each sample was determined.

### X-gal Quantification of Cardiac-MHC Rich Areas

Embryoid bodies for each experimental condition were fixed with glutaraldehyde (Sigma-Aldrich) for 30 min at room temperature, followed by a 30 min wash with phosphate-based buffer and overnight incubation at 37°C with X-gal staining solution (Invitrogen), as previously described [62]. Embryoid body images were captured with a ProgRes C3 camera-equipped Zeiss stereo Discovery V20 microscope.

### Statistics

Values are provided as mean 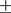 SE and Student's *t*-tests with 95% confidence intervals were used to compare treatment groups. p<0.05 was predetermined as significant.

## References

[1] Rosenthal N (2003). Prometheus's vulture and the stem-cell promise.. N Engl J Med.

[2] Slack JM (2008). Origin of stem cells in organogenesis.. Science.

[3] Gurdon JB, Melton DA (2008). Nuclear reprogramming in cells.. Science.

[4] Murry CE, Keller G (2008). Differentiation of embryonic stem cells to clinically relevant populations: lessons from embryonic development.. Cell.

[5] Nelson TJ, Behfar A, Terzic A (2008). Stem cells: biologics for regeneration.. Clin Pharmacol Ther.

[6] Graf T, Enver T (2009). Forcing cells to change lineages.. Nature.

[7] Nelson TJ, Behfar A, Yamada S, Martinez-Fernandez A, Terzic A (2009). Stem cell platforms for regenerative medicine.. Clin Transl Sci.

[8] Passier R, van Laake LW, Mummery CL (2008). Stem-cell-based therapy and lessons from the heart.. Nature.

[9] Jaenisch R, Young R (2008). Stem cells, the molecular circuitry of pluripotency and nuclear reprogramming.. Cell.

[10] Muller FJ, Laurent LC, Kostka D, Ulitsky I, Williams R (2008). Regulatory networks define phenotypic classes of human stem cell lines.. Nature.

[11] Arrell DK, Niederlander NJ, Faustino RS, Behfar A, Terzic A (2008). Cardioinductive network guiding stem cell differentiation revealed by proteomic cartography of tumor necrosis factor α-primed endodermal secretome.. Stem Cells.

[12] Christoforou N, Miller RA, Hill CM, Jie CC, McCallion AS (2008). Mouse ES cell-derived cardiac precursor cells are multipotent and facilitate identification of novel cardiac genes.. J Clin Invest.

[13] Laflamme MA, Chen KY, Naumova AV, Muskheli V, Fugate JA (2007). Cardiomyocytes derived from human embryonic stem cells in pro-survival factors enhance function of infarcted rat hearts.. Nat Biotechnol.

[14] Braam SR, Passier R, Mummery CL (2009). Cardiomyocytes from human pluripotent stem cells in regenerative medicine and drug discovery.. Trends Pharmacol Sci.

[15] Laflamme MA, Murry CE (2005). Regenerating the heart.. Nat Biotechnol.

[16] Faustino RS, Behfar A, Perez-Terzic C, Terzic A (2008). Genomic chart guiding embryonic stem cell cardiopoiesis.. Genome Biol.

[17] Moretti A, Caron L, Nakano A, Lam JT, Bernshausen A (2006). Multipotent embryonic Isl1+ progenitor cells lead to cardiac, smooth muscle, and endothelial cell diversification.. Cell.

[18] Kattman SJ, Huber TL, Keller GM (2006). Multipotent Flk-1^+^ cardiovascular progenitor cells give rise to the cardiomyocyte, endothelial, and vascular smooth muscle lineages.. Dev Cell.

[19] Nelson TJ, Chiriac A, Faustino RS, Crespo-Diaz RJ, Behfar A (2009). Lineage specification of Flk-1^+^ progenitors is associated with divergent Sox7 expression in cardiopoiesis.. Differentiation.

[20] Segers VF, Lee RT (2008). Stem-cell therapy for cardiac disease.. Nature.

[21] Wu SM, Chien KR, Mummery C (2008). Origins and fates of cardiovascular progenitor cells.. Cell.

[22] Olson EN (2006). Gene regulatory networks in the evolution and development of the heart.. Science.

[23] Horton RE, Millman JR, Colton CK, Auguste DT (2009). Engineering microenvironments for embryonic stem cell differentiation to cardiomyocytes.. Regen Med.

[24] Behfar A, Faustino RS, Arrell DK, Dzeja PP, Perez-Terzic C (2008). Guided stem cell cardiopoiesis: discovery and translation.. J Mol Cell Cardiol.

[25] Srivastava D, Olson EN (2000). A genetic blueprint for cardiac development.. Nature.

[26] Urbanek K, Cesselli D, Rota M, Nascimbene A, De Angelis A (2006). Stem cell niches in the adult mouse heart.. Proc Nat Acad Sci USA.

[27] Behfar A, Perez-Terzic C, Faustino RS, Arrell DK, Hodgson DM (2007). Cardiopoietic programming of embryonic stem cells for tumor-free heart repair.. J Exp Med.

[28] Nelson TJ, Faustino RS, Chiriac A, Crespo-Diaz R, Behfar A (2008). CXCR4^+^/Flk-1^+^ biomarkers select a cardiopoietic lineage from embryonic stem cells.. Stem Cells.

[29] Arias AM, Hayward P (2006). Filtering transcriptional noise during development: concepts and mechanisms.. Nat Rev Genet.

[30] Chang HH, Hemberg M, Barahona M, Ingber DE, Huang S (2008). Transcriptome-wide noise controls lineage choice in mammalian progenitor cells.. Nature.

[31] Pickart MA, Klee EW, Nielsen AL, Sivasubbu S, Mendenhall EM (2006). Genome-wide reverse genetics framework to identify novel functions of the vertebrate secretome.. PloS One.

[32] Ulloa-Montoya F, Kidder BL, Pauwelyn KA, Chase LG, Luttun A (2007). Comparative transcriptome analysis of embryonic and adult stem cells with extended and limited differentiation capacity.. Genome Biol.

[33] Brandenberger R, Wei H, Zhang S, Lei S, Murage J (2004). Transcriptome characterization elucidates signaling networks that control human ES cell growth and differentiation.. Nat Biotechnol.

[34] Faustino RS, Chiriac A, Terzic A (2008). Bioinformatic primer for clinical and translational science.. Clin Transl Sci.

[35] Lu R, Markowetz F, Unwin RD, Leek JT, Airoldi EM (2009). Systems-level dynamic analyses of fate change in murine embryonic stem cells.. Nature.

[36] Barabasi AL, Oltvai ZN (2004). Network biology: understanding the cell's functional organization.. Nat Rev Genet.

[37] Raser JM, O'Shea EK (2004). Control of stochasticity in eukaryotic gene expression.. Science.

[38] Raser JM, O'Shea EK (2005). Noise in gene expression: origins, consequences, and control.. Science.

[39] Swain PS, Elowitz MB, Siggia ED (2002). Intrinsic and extrinsic contributions to stochasticity in gene expression.. Proc Natl Acad Sci USA.

[40] Faustino RS, Terzic A (2008). Interactome of a cardiopoietic precursor.. J Cardiovas Transl Res.

[41] Chien KR, Domian IJ, Parker KK (2008). Cardiogenesis and the complex biology of regenerative cardiovascular medicine.. Science.

[42] Kwon C, Qian L, Cheng P, Nigam V, Arnold J (2009). A regulatory pathway involving Notch1/beta-catenin/Isl1 determines cardiac progenitor cell fate.. Nat Cell Bio.

[43] Beqqali A, Kloots J, Ward-van Oostwaard D, Mummery C, Passier R (2006). Genome-wide transcriptional profiling of human embryonic stem cells differentiating to cardiomyocytes.. Stem Cells.

[44] Cao F, Wagner RA, Wilson KD, Xie X, Fu JD (2008). Transcriptional and functional profiling of human embryonic stem cell-derived cardiomyocytes.. PloS One.

[45] Leschik J, Stefanovic S, Brinon B, Puceat M (2008). Cardiac commitment of primate embryonic stem cells.. Nat Protoc.

[46] Rajasingh J, Bord E, Hamada H, Lambers E, Qin G (2007). STAT3-dependent mouse embryonic stem cell differentiation into cardiomyocytes: analysis of molecular signaling and therapeutic efficacy of cardiomyocyte precommitted mES transplantation in a mouse model of myocardial infarction.. Circ Res.

[47] Chen Y, Amende I, Hampton TG, Yang Y, Ke Q (2006). Vascular endothelial growth factor promotes cardiomyocyte differentiation of embryonic stem cells.. Am J Physiol Heart Circ Physiol.

[48] Coultas L, Chawengsaksophak K, Rossant J (2005). Endothelial cells and VEGF in vascular development.. Nature.

[49] Nagasawa T, Hirota S, Tachibana K, Takakura N, Nishikawa S (1996). Defects of B-cell lymphopoiesis and bone-marrow myelopoiesis in mice lacking the CXC chemokine PBSF/SDF-1.. Nature.

[50] Penn MS (2009). Importance of the SDF-1:CXCR4 axis in myocardial repair.. Circ Res.

[51] Chung S, Dzeja P, Faustino R, Terzic A (2008). Developmental restructuring of the creatine kinase system integrates mitochondrial energetics with stem cell cardiogenesis.. Ann NY Acad Sci.

[52] Chung S, Dzeja PP, Faustino RS, Perez-Terzic C, Behfar A (2007). Switch in energy metabolism with mitochondria network maturation supports cardiac differentiation of stem cells.. Nat Clin Pract Cardiovasc Med.

[53] Arrell DK, Niederlander NJ, Perez-Terzic C, Chung S, Behfar A (2007). Pharmacoproteomics: advancing the efficacy and safety of regenerative therapeutics.. Clin Pharmacol Ther.

[54] Hodgson DM, Behfar A, Zingman LV, Kane GC, Perez-Terzic C (2004). Stable benefit of embryonic stem cell therapy in myocardial infarction.. Am J Physiol Heart Circ Physiol.

[55] Robey TE, Saiget MK, Reinecke H, Murry CE (2008). Systems approaches to preventing transplanted cell death in cardiac repair.. J Mol Cell Cardiol.

[56] Perez-Terzic C, Behfar A, Mery A, van Deursen JM, Teric A (2003). Structural adaptation of the nuclear pore complex in stem cell-derived cardiomyocytes.. Circ Res.

[57] Perez-Terzic C, Faustino RS, Boorsma BJ, Arrell DK, Niederlander NJ (2007). Stem cell transformation into cardiac phenotype guided by direct remodeling of nuclear transport machinery.. Nat Clin Pract Cardiovasc Med.

[58] Arell DK, Zlatkovic J, Kane GC, Yamada S, Terzic A (2009). ATP-sensitive K^+^ channel knockout induces cardiac proteome remodeling predictive of heart disease susceptibility.. J Proteome Res.

[59] Zlatkovic J, Arell DK, Kane GC, Miki T, Seino S, Terzic A (2009). Proteomic profiling of K_ATP_ channel deficient hypertensive heart mapps risk for maladaptive cardiomyopathic outcome.. Proteomics.

[60] Assenov Y, Ramirez F, Schelhorn SE, Lengauer T, Albrecht M (2008). Computing topological parameters of biological networks.. Bioinformatics.

[61] Misra RP, Bronson SK, Xiao Q, Garrison W, Li J (2001). Generation of single-copy transgenic mouse embryos directly from ES cells by tetraploid embryo complementation.. BMC Biotechnol.

[62] Nelson TJ, Ge ZD, Van Orman J, Barron M, Rudy-Reil D (2006). Improved cardiac function in infarcted mice after treatment with pluripotent embryonic stem cells.. Anat Rec A Discov Mol Cell Evol Biol.

[63] Veldkamp CT, Ziarek JJ, Su J, Basnet H, Lennertz R (2009). Monomeric structure of the cardioprotective chemokine SDF-1/CXCL12.. Protein Sci.

[64] Germani A, Di Carlo A, Mangoni A, Straino S, Giacinti C (2003). Vascular endothelial growth factor modulates skeletal myoblast function.. Am J Pathol.

[65] Huang F, He J, Zhang Y, Guo Y (2008). Synthesis of biotin-AMP conjugate for 5′ biotin labeling of RNA through one-step in vitro transcription.. Nat Protoc.

